# Properties and temporal dynamics of choice- and action-predictive signals during item recognition decisions

**DOI:** 10.1007/s00429-020-02124-4

**Published:** 2020-08-09

**Authors:** Roberto Guidotti, Annalisa Tosoni, Carlo Sestieri

**Affiliations:** Department of Neuroscience, Imaging and Clinical Sciences, ITAB Institute for Advanced Biomedical Technologies, G. D’Annunzio University of Chieti-Pescara, 66100 Chieti, Italy

**Keywords:** Episodic memory, Item recognition, Decision-making, Motor intention, MVPA

## Abstract

Decision-making is in the service of action regardless of whether the decision concerns perceptual information, goods or memories. Compared to recent advances in the neurobiology of perceptual or value-based decisions, however, the neural bases supporting the sampling of evidence in long-term memory, and the transformation of memory-based decisions into appropriate actions, are still poorly understood. In the present fMRI study, we used multivariate pattern analysis to investigate the temporal dynamics of choice- and action-predictive signals during an item recognition task that manipulated the association between memory choices (old/new) and motor responses (eye/hand) across subjects. Choice-predictive activity was mainly observed in striatal, lateral prefrontal and lateral parietal regions, was sensitive to the amount of decision evidence and showed a rapid increase after stimulus onset, followed by a fast decay. Action-predictive signals were found in primary sensory motor, premotor and occipito–parietal regions, were generally observed at the end of the decision phase and were not modulated by decision evidence. These findings suggest that a memory decision variable, potentially represented in a fronto–striato–parietal network, is not directly transformed into an action plan as often observed in perceptual decisions. Regions exhibiting choice predictive activity, and especially the striatum, however, also showed a second peak of decision-related activity that, unlike pure choice- or action-predictive signals, depended on the particular choice–response association. This second peak of activity in the striatum might represent the neural signature of the transformation of a memory decision into an appropriate motor response based on the specific choice–response association.

**Electronic supplementary material:**

The online version of this article (10.1007/s00429-020-02124-4) contains supplementary material, which is available to authorized users.

## Introduction

Decision-making involves the evaluation of evidence for a particular choice and the selection of an appropriate action (Kable and Glimcher 2009; Shadlen and Shohamy 2016). While perceptual and value-based decisions are emblematic of this strong sensori-motor chain, the association between decisions and actions can be less evident in other contexts. However, also decisions based on information stored in long-term memory can directly inform actions, such as when we orient our gaze toward someone that speaks with a familiar voice. In general, it is often argued that a main evolutionary advantage of a neural system dedicated to remember past episodes is to mentally represent possible scenarios and guide future behaviors (Suddendorf and Corballis 2007). While extensive research has been conducted to uncover the neural mechanisms underlying perceptual (Gold and Shadlen 2007; Heekeren et al. 2008) and value-based decisions (Rangel et al. 2008; Sugrue et al. 2005) the neural bases supporting the sampling of evidence in long-term memory and the transformation of memory-based decisions into appropriate actions are still underspecified.

The first clue about the neural mechanisms involved in memory-based decisions in humans has been the observation that BOLD activity in parietal (Kahn et al. 2004; Wheeler and Buckner 2003), but also striatal (Abe et al. 2008), regions during old/new recognition judgments better tracks the subjective feeling of oldness, rather than the objective memory status. Based on these early findings and other empirical observations (e.g. Sestieri et al. 2011), it has been proposed that some regions of the posterior parietal cortex (PPC) might serve as a mnemonic accumulator of evidence for recognition decisions (Sestieri et al. 2017; Wagner et al. 2005), in analogy with the accumulation of sensory information during perceptual decisions (Gold and Shadlen 2007). This hypothesis is grounded on the seminal theoretical work by Ratcliff and McKoon, who proposed that also the simple act of judging whether an item is old or new can be conceptualized in a decision-making framework (Ratcliff 1978). From this perspective, item recognition judgments are performed on the basis of a decision-to-bound, or evidence accumulation, process determined by the relatedness between the probe and the items present in the memory set. While the resonance between features of the probe and items within the set represents evidence for old decisions, new decisions are made when the search for relatedness terminates in a non-match (Ratcliff 1978). Consistent with this model, we have subsequently identified BOLD signals compatible with the representation of a decision variable during item recognition (Sestieri et al. 2014) and source memory (Guidotti et al. 2019) decisions in regions located within or adjacent to the intraparietal sulcus (IPS). Again, these signals appear to reflect the outcome of a decision process as they were significantly modulated by decision evidence and were predictive of the subject’s choices even when task performance was almost at chance.

Based on its traditional role in action preparation and execution across different response effectors (Andersen and Cui 2009), the PPC also appears ideally suited for transforming mnemonic evidence into appropriate actions. In our previous study (Sestieri et al. 2014), we have tested the hypothesis that a memory decision variable is directly encoded in parietal regions involved in movement planning, in analogy to what has been shown in several electrophysiological (Gold and Shadlen 2007; Shadlen and Newsome 2001) and neuroimaging (Donner et al. 2009; Gould et al. 2012; Tosoni et al. 2008, 2014) studies on perceptual decisions. Surprisingly, we found that signals for memory-based decisions and motor intentions were largely independent: effector-preferring regions of the PPC were not modulated by decisions evidence, while regions that tracked evidence for oldness did so regardless of the motor effector that was associated with the choice. While this finding suggests a substantial segregation between the neural systems involved in perceptual and memory-based decisions (Sestieri et al. 2017), the question of how memory decisions are transformed into appropriate motor plans remains unaddressed.

An additional candidate region for implementing the transformation of memory decisions into motor actions is the striatum. The basal ganglia have been traditionally associated with the expression of non-declarative memory, but increasing evidence from human studies have indicated an additional role in different aspects of declarative memory performance [reviewed in Scimeca and Badre (2012)]. Importantly, the striatum is not conceived as the source of mnemonic signals, but likely encodes a set of modulatory signals representing the value of the retrieved information in the service of the current goals (Bunzeck et al. 2010; Han et al. 2010). Beyond a direct role in the evaluation process, moreover, classical theories of striatal functions highlight its crucial involvement in the creation of the optimal stimulus–response associations (e.g. Sugrue et al. 2005) and in the selection of appropriate responses (e.g. see Redgrave et al. 2010; Redgrave et al. 2011). Therefore, through its diffuse pattern of connections with the neocortex, the striatum appears perfectly suited to implement and monitor the correct association between memory decisions and the expression of specific actions.

In the present work, we leverage the strong sensitivity of multivariate pattern analysis (MVPA) (King and Dehaene 2014; Kriegeskorte et al. 2006; Rissman and Wagner 2012; Tong and Pratte 2012) to investigate the dynamics of choice- and action-predictive signals in the human brain and shed light on the potential mechanisms mediating the sensori-motor transformation during memory-based decisions. We reanalyzed the data from our previous study on item recognition decisions (Sestieri et al. 2014), in which a choice (old vs. new decision)/response (hand vs. eye movement) association was manipulated across subjects to dissociate the representation of a decision variable from the preparation of the motor response. We first identified brain regions where the locally distributed pattern of activity encoded memory choices and motor intentions across subjects. Next, we examined whether choice- and action-predictive activity were modulated by the amount of decisions evidence. While we expect choice-predictive activity to positively scale with decision certainty (see Guidotti et al. 2019), a similar effect on action-predictive activity would indicate that regions involved in action planning can directly encode a decision variable. We further tracked the temporal profile of choice- and action-predictive activity to test whether the two decoding signals increased simultaneously or showed a distinct temporal profile. Finally, we compared the temporal profile of decoding accuracy of a within- and a between-subject analysis to investigate potential neural mechanisms underlying the transformation of a memory decision into an appropriate response based on the specific choice–response association.

## Methods

This article is based on a new analysis of a previously published experiment (Sestieri et al. 2014; Tosoni et al. 2016). Because stimuli, tasks and procedures have been extensively described in these publications; here, we present a summary description.

### Participants

A group of 24 right-handed subjects (11 males, mean age 25 ± 3 years) participated in the experiment after giving informed consent in accordance with guidelines set by the Human Studies Committee of G. D’Annunzio Chieti University. The memory experiment involved two sessions: a behavioral encoding session performed in a testing room followed by a retrieval session inside the MRI scanner. The interval between the encoding and the retrieval sessions was approximately 24 h.

### Stimuli

Stimuli consisted of 256 by 256 pixel color photographs depicting indoor and outdoor scenes, selected from a large database [(Konkle et al. 2010), https://www.cvcl.mit.edu/MM]. A total of 484 images (64 for practice, 420 for the experiment) were used. Visual stimuli were presented using E-Prime 1.1 software (Psychology Software Tools).

### Apparatus

The encoding session was performed inside a mock scanner. In both sessions, stimuli were projected onto a screen positioned at the back of the scanner via a LCD projector and visible to subjects through a mirror attached over the subject’s head. Subjects responded using a Cedrus RB-830 USB Response Pad and Cedrus Lumina LU400 fiber optic Response Pad in the encoding and retrieval session, respectively.

### Procedure

#### Rationale of evidence manipulation

A main variable that affects decision-making is the amount of information favoring one of the possible choice options, defined as decision evidence. In the present study, the amount of evidence favoring old decisions was manipulated by varying the “encoding strength”, defined as the number of item repetition during the encoding session. The rationale was that items repeated more times should be associated with more evidence favoring “old” decisions and thus, higher accuracy and faster decision times. The amount of evidence favoring new decisions was instead manipulated by varying the similarity between old and new item images under the rationale that highly distinctive (i.e. less similar) retrieval lures should be associated with greater evidence for a “new” decision.

#### Encoding session

At encoding, subjects made indoor/outdoor decisions on visually presented images depicting scenes from different categories. Images from 60 categories (30 indoor and 30 outdoor) were presented, each comprising 4 different stimuli, resulting in a total of 240 images. The four stimuli in each category were presented with different frequencies: two images were presented once [“1 ×” and encoding only (“EO”) images], one was presented three times (“3 ×”) and one was presented five times (“5 ×”). A total of 15 blocks, each including 4 1 ×, 4 EO, 12 3 ×, and 20 5 × stimuli, were presented. Each trial started with a 500 ms warning red fixation cross on a gray background, followed by image presentation for 1 s. The image was followed by a 2 s blue fixation cross. Subjects had a total of 3 s from the image onset to provide a response on the indoor/outdoor discrimination task by pressing one of two keys of a response pad located under their right hand. A white fixation cross preceded the next trial.

#### Retrieval session

Approximately 24 h later, subjects were involved in an item recognition decision task. Old items (*N* = 180) included the whole set of 1 ×, 3 ×, and 5 × images presented at encoding, while EO images were only used to manipulate the perceptual similarity between old and new items and thus were not presented at retrieval. Three types of new items (*N* = 180) were presented, characterized by a decreasing evidence level toward new decisions: images belonging to 60 new categories [unrelated (“U”) to old images], images belonging to the same 60 encoding categories (semantically related, “SR”), and images that were both semantically and perceptually similar to EO images (“SPR”). A total of 12 blocks, each including 30 trials, 5 for each of the 6 retrieval stimulus types, were presented. Each trial started with the presentation of an image for 1.5 s along with a left or right peripheral target (white circle). An 8 s delay preceded the go-signal for the execution of either a saccade or a pointing movement toward the remembered peripheral target. The association between the memory choice (old/new) and the motor response (hand/eye movement) was provided at the beginning of the experiment and was counterbalanced across groups (*N* = 12 each). The across-subjects manipulation of the choice–response association was conducted to dissociate the neural signals associated with the memory decision from those associated with the motor intention/preparation. A variable inter-stimulus interval (2–4 MR frames) preceded the next trial. Participants performed a total of 360 trials divided in 12 runs.

### Behavioral data analysis

A three-way ANOVA with memory status (old, new) and evidence (low, middle, high) as the within-subject factors and choice–response association (A1, A2) as the between-subject factor was conducted to test whether item recognition performance significantly increased as a function of decision evidence. Duncan tests were used for post hoc analyses. While the present fMRI paradigm made use of a fixed delay between the onset of the stimulus and the motor response, the analysis of the behavioral data from a reaction time version of the experiment demonstrated that the current manipulation specifically affects the process of evidence accumulation in a diffusion model framework (see Sestieri et al. 2014 for details about the analysis and results of this supplemental experiment).

### fMRI scanning parameters

Functional T2*-weighted images were collected on a Philips Achieva 3T scanner, using a gradient-echo EPI sequence to measure the BOLD contrast over the whole brain (TR = 1914 ms, TE = 25 ms, 39 slices acquired in ascending interleaved order, voxel size 3.59 × 3.59 × 3.59 mm, 64 × 64 matrix, flip angle = 80°). Structural images were collected using a sagittal FFE T1-weighted sequence (TR = 8.14 ms, TE = 3.7 ms, flip angle = 8°, voxel size = 1 × 1 × 1 mm) and a T2-weighted sequence (TR = 3 s, TE = 80 ms, flip angle = 90°, voxel size = 0.98 × 1 × 1 mm, 39 slices).

### fMRI preliminary data analysis

Preprocessing and data analysis were performed using an in-house software (fIDL) developed at Washington University in St. Louis. BOLD images were motion-corrected within and between runs, corrected for across-slice timing differences, resampled into 3 mm isotropic voxels, and warped into 711-2C space, a standardized atlas space (Van Essen 2005). Preprocessing included a whole-brain normalization correcting for changes in overall image intensity between BOLD runs.

### Multivariate pattern analyses

We used MVPA (Haynes 2015; Haynes and Rees 2006) to understand whether the locally and temporally distributed pattern of activity carried information about different task-related information (memory choices, motor response, target side and image type). Analyses were performed using *nilearn* (Abraham et al. 2014), *pymvpa* (Hanke et al. 2009), *MNE* (Gramfort et al. 2013) and *scipy* (Oliphant 2007).

#### General linear models and datasets construction

We first modeled and removed the BOLD activity associated with movement execution time-locked to the onset of the go-signal. Hemodynamic responses associated with the decision and the motor execution phases were estimated using a standard GLM approach with assumption of the hemodynamic response function. The model included 12 regressors starting at the onset of the image [memory status (old, new); evidence (high, middle, low); accuracy (correct, incorrect)] and 4 regressor starting at the onset of the go-signal [motor response (eye, hand), accuracy (correct, incorrect)]. The assumed response for each process was generated by convolving a rectangle function representing the duration of the process (1.5 s for the decision phase corresponding to image duration, 1 s for execution phase corresponding to go-signal duration) with a standard hemodynamic response function [HRF, (Boynton et al. 1996)].

Next, we obtained a residual dataset which retained the BOLD activity associated with the decision phase, while removing the activity associated with the execution phase. The resulting dataset was directly used for regional multivariate analyses that treated BOLD activity at the level of individual MR frames (temporal MVPA). To increase the sensitivity of the MVPA searchlight analyses looking for choice-predictive and action-predictive signals, we created an additional linear model that included a regressor for each individual trial starting at image onset. Single-trial betas were extracted by assuming a shape of the HRF (Mumford et al. 2012).

#### Between-subject searchlight MVPA

We performed searchlight analyses (Kriegeskorte and Bandettini 2007) to identify brain regions in which locally distributed activity predicted memory choices and motor responses. Since the association between the memory choice and the motor response was fixed for each subject, the patterns associated with these two processes are perfectly collinear and cannot be distinguished using a standard within-subject classification analysis. However, since the choice–response association was counterbalanced across subjects, we employed a between-subject design to disentangle choice- from action-predictive signals. As shown in our previous work on classification of source memory decision signals (Guidotti et al. 2019), this approach exploits the reversal of the choice/response association across subjects to identify signals that uniquely predict the memory decision or the motor response. Therefore, the between-subject searchlight approach identifies voxels in which choice-predictive (or action-predictive) locally distributed activity is shared across subjects, regardless of the particular association. This approach is, therefore, insensitive to signals that are specific for a particular subject (i.e. choice–response association).

Two searchlight MVPA analyses investigated the spatial distribution of signals related to additional task-related processes during the decision phase of the task: the subcategory of the scene image (image type: outdoor, indoor) and the target location for the upcoming movement (target side: left, right). The first control analysis was conducted to test the ability of the between-subject decoding approach to identify brain regions associated with scene perception [e.g. parahippocampal place area, retrosplenial cortex (Epstein 2008)] independently of the memory decision. The second analysis aimed to identify locally distributed activity that distinguished between left and right peripheral targets. In this case, successful decoding of target side would reflect a combination of two different signals: a transient perceptual/attentional signal associated with the lateralized presentation of the target stimulus and a more sustained sensorimotor signal associated with the lateralized component of the motor intention.

The searchlight analyses were carried out using the single-trial beta dataset as input for the four classification analyses. We normalized input maps using *z* score to remove the overall level of the BOLD activity. This procedure ensured that the classification did not rely on average signal differences across conditions, but on the spatial distribution of activity (Davis et al. 2014; Hebart and Baker 2017). Finally, we detrended and normalized using *z* score voxel activity across trials. We scrolled a sphere of 3 voxel radius (up to 123 voxels/sphere) across the brain maps. For each sphere, a linear support vector machine (SVM) with regularization parameter *C* = 1 was trained and tested in a leave-one-subject-out cross-validation approach, assigning the testing accuracy to each sphere center (Etzel et al. 2013). Since the leave-one-subject-out searchlight is computationally demanding, we randomly removed, in each subject, 50% of the trials, keeping the dataset balanced between conditions. A voxelwise paired t test versus chance level (accuracy = 50%) was used to evaluate statistical significance of the accuracy maps, then we applied a FDR (*α* = 0.01) and a cluster-level thresholding (cluster size = 50 voxel) to isolate clusters of significant voxels.

#### Effect of decision evidence on regional decoding

Regions of interest (ROIs) identified through the searchlight analysis were used in subsequent regional analyses to examine whether classification accuracy was modulated by decision evidence (i.e. evidence favoring a particular memory choice). We ran a different classification analysis for each task-related information and amount of decision evidence (low, middle, high) with the same analysis pipeline of the searchlight approach. To increase sensitivity, we used an ANOVA-based feature selection (Pereira et al., 2009) by selecting the first *k* = 50 highest *f* score rank features for each cluster. Feature selection was only applied on the training set to avoid biasing of the classification error (Pereira et al. 2009). Importantly, feature selection also ensured that classification accuracy was not biased by cluster size. We used a linear mixed effect model (Chen et al. 2013) to test for the linear effect of a covariate represented by the amount of decision evidence. An additional analysis focused on the low-evidence condition in which the subjective choice and the objective memory status were maximally divergent and performance was near chance. First, a one-sample *t* test versus chance level (accuracy = 50%) was used to test the presence of significant classification accuracy. Furthermore, a paired *t* test (two-tailed) was used to assess whether choice-classification was greater than classification of the memory status). In the latter case, old and new labels refer to the objective, rather than perceived, oldness and newness of the item. Statistical values were Bonferroni corrected by the number of ROIs identified in each searchlight analysis.

#### Regional temporal decoding

We also characterized the temporal profile of choice- and action-predictive signals by extracting the time-course of activity associated with the first seven MR frames (i.e. time-points) following image presentation for each trial and ROI defined in the searchlight analysis, using the residual dataset (see paragraph general linear models and datasets construction). The epoch corresponds to the entire delay period plus an additional MR frame belonging to the execution phase. For each time point, we trained a linear SVM with *C* = 1 and an ANOVA-based feature selection with *k* = 50 and we tested the presence of significant classification accuracy, obtaining a time course of classification accuracy. Statistical values were Bonferroni corrected by the number of time points times the number of ROIs identified in the corresponding searchlight analysis. We note that this analysis is expected to have less power than the analysis conducted on single trial betas for several reasons: higher sensitivity of models that assume a shape of the HRF, potential inter-individual differences in the time course of classification accuracy, higher number of comparisons that impact on Bonferroni correction.

#### Temporal within-subject decoding and temporal generalization analyses

The regional temporal decoding analyses were also performed using a within-subject approach. Our rationale was that the comparison of the between- and the within-subject approach can help identifying brain regions in which decision-related activity (either choice- or action-predictive) is dependent on the particular choice/response association. Thus, while the within-subject decoding cannot distinguish between choice- and action-predictive activity, as they are perfectly collinear in each subject, it can additionally identify idiosyncratic decision-related activity that reflects the specific choice–response association.

The within-subject temporal decoding followed the same pipeline of the previous analysis but employed a classifier that was trained separately for each subject using a *k*-fold leave-onefold-out cross-validation (*k* = 7) approach. Moreover, we used the temporal generalization method developed by King and Dehaene (2014) to test whether a classifier trained at a specific time point performed above-chance also at other time-points. We obtained an accuracy matrix indicating how the pattern generalizes across-time or, in other words, whether the distributed pattern of activity was stable or changed significantly during the trial. Significant across-time point decoding was tested by means of a one-sample *t* test versus chance level (accuracy = 50%) and a Bonferroni correction by the number of elements in each matrix (*N* = 49) multiplied by the number of ROIs.

## Results

### Behavioral results

As expected, the manipulation of memory evidence had a robust effect on recognition accuracy. The three-way ANOVA with memory status (old, new), evidence (low, middle, high) and choice–response association (A1, A2) as factors revealed a robust main effect of evidence [*F*(2, 44) = 275.0, *p* < 0.0001]. Post hoc tests indicated the presence of a significant difference across the three levels (low: 0.57 ± 0.01, middle: 0.75 ± 0.01, high: 0.87 ± 0.01; all tests significant at *p* < 0.0001], confirming that performance increased gradually as a function of decision evidence. The ANOVA further revealed a significant main effect of memory status [*F*(1, 22) = 6.7, *p* < 0.05)], indicating better performance for new (0.76 ± 0.02) vs. old (0.70 ± 0.01) items, and a significant evidence by memory status interaction [*F*(2, 44) = 20.0, *p* < 0.0001]. Post hoc tests showed that the interaction was explained by a better performance for new versus old items at the lowest level of evidence [0.65 ± 0.02 vs. 0.50 ± 0.02, *p* < 0.0001]. No other effect of the ANOVA was significant. Importantly, the lack of a significant main effect of choice–response association, and of any interaction effect involving this factor, indicates a comparable performance across the two groups.

### Spatial distribution of choice-predictive and action-predictive activity

As a first step, we used a searchlight analysis to identify brain regions whose locally distributed activity predicted different task-related processes, i.e. memory choices, motor response, target side and image type. The identified clusters that were used as ROIs in subsequent analysis are presented in Supplemental Table 1).

**Fig. 1:**
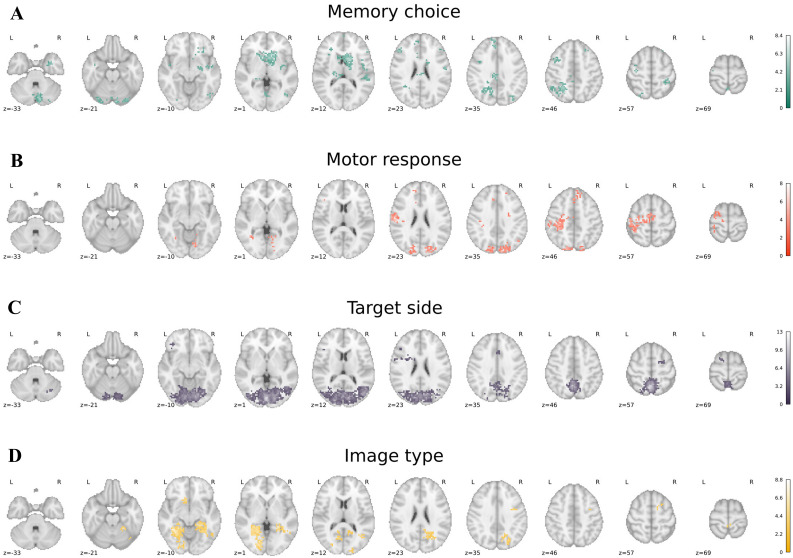
Searchlight maps of task-related processes. The figure shows the significant clusters of locally distributed activity associated with memory choice (first row; green), motor response (second row, red), target side (third row, purple) and image type (fourth row, yellow)

The largest clusters of voxels exhibiting choice-predictive activity (Fig. 1a) were centered on the right caudate nucleus, the left lateral PPC, the left lateral prefrontal cortex (PFC) and the cerebellum (Fig. 1, first row). The location of these clusters closely matches the location of regions that were associated with memory decision-making in our previous work (Sestieri et al. 2014), despite the two approaches focused on different indices of BOLD activity during the decision phase (spatially distributed versus averaged activity, choice-predictive versus parametric modulation of memory evidence, etc.).

As expected, the searchlight map of action-predictive activity (i.e. motor response) included a large cluster located in the sensori-motor and premotor cortex along the central sulcus, including parts of the precentral/postcentral gyri (Fig. 1b). These clusters were located exclusively on the left hemisphere, with the exception of the supplementary motor cortex. These results, obtained through a data-driven approach during the decision phase (i.e. before movement execution), corroborate the findings obtained in our original work using univariate analyses on individually defined ROIs (Sestieri et al. 2014). Bilateral clusters were also found in portions of cortex located along the parieto-occipital sulcus and the adjacent dorsal occipital cortex. These regions are more ventral than the individually defined parietal ROIs that were employed in our previous study and appear to partially correspond to regions V6 and V6A ventral (Pitzalis et al. 2015; Tosoni et al. 2015). The discrepancy potentially reflects the present focus on activity that generalized across subjects rather than on individuals. Another large cluster was identified in the right ventral visual cortex. The presence of action-predictive activity in the ventral visual cortex is consistent with the observation of effector-specificity in similar regions during the response phase of the task (Tosoni et al. 2016).

Locally distributed activity associated with target side was mainly identified in a large bilateral cluster spanning the medial and lateral visual cortex and the medial parietal cortex (Fig. 1c). However, the current design does not allow to distinguish whether these patterns were more associated with the perception of the peripheral target stimulus or with a lateralized component of action intention/planning. The identified cluster likely represents a variable mixture of the two signals. Finally, as expected, large clusters of voxels that differentiated between indoor and outdoor scenes were found in bilateral posterior regions corresponding to the parahippocampal place area and the retrosplenial cortex (Fig. 1d), supporting the representation of sub-categories of real-world scenes in these regions (Epstein and Higgins 2007).

### Selective effect of decision evidence on choice-predictive activity

We further tested whether the identified clusters of locally distributed activity associated with memory choice, motor response, target side and image type were modulated by the amount of evidence for the memory choice, which had a robust, parametric effect on behavioral performance. We expected choice-predictive activity to scale with the amount of decision evidence, reflecting the increasing difference in the local spatial distribution of activity for the two outcomes (Guidotti et al. 2019). In contrast, as information about the subcategory of the presented images (indoor, outdoor) was likely only relevant for the encoding session, we expected no effect of this variable on classification accuracy associated with image type. As far as the modulation of decision evidence on action-predictive (i.e. motor response) activity, a significant effect could be expected if decision evidence were directly translated in action-related signals, in accordance with an intentional framework (Shadlen et al. 2008; Tosoni et al. 2008, 2014). However, a marginal or null effect of this variable is expected based on our previous univariate findings of no significant BOLD modulation of memory evidence in sensorimotor regions associated with the planning of the motor response (Sestieri et al. 2014). Thus, this analysis tested whether a link between decision evidence and motor intentions can be found using a more sensitive multivariate approach.

**Fig. 2:**
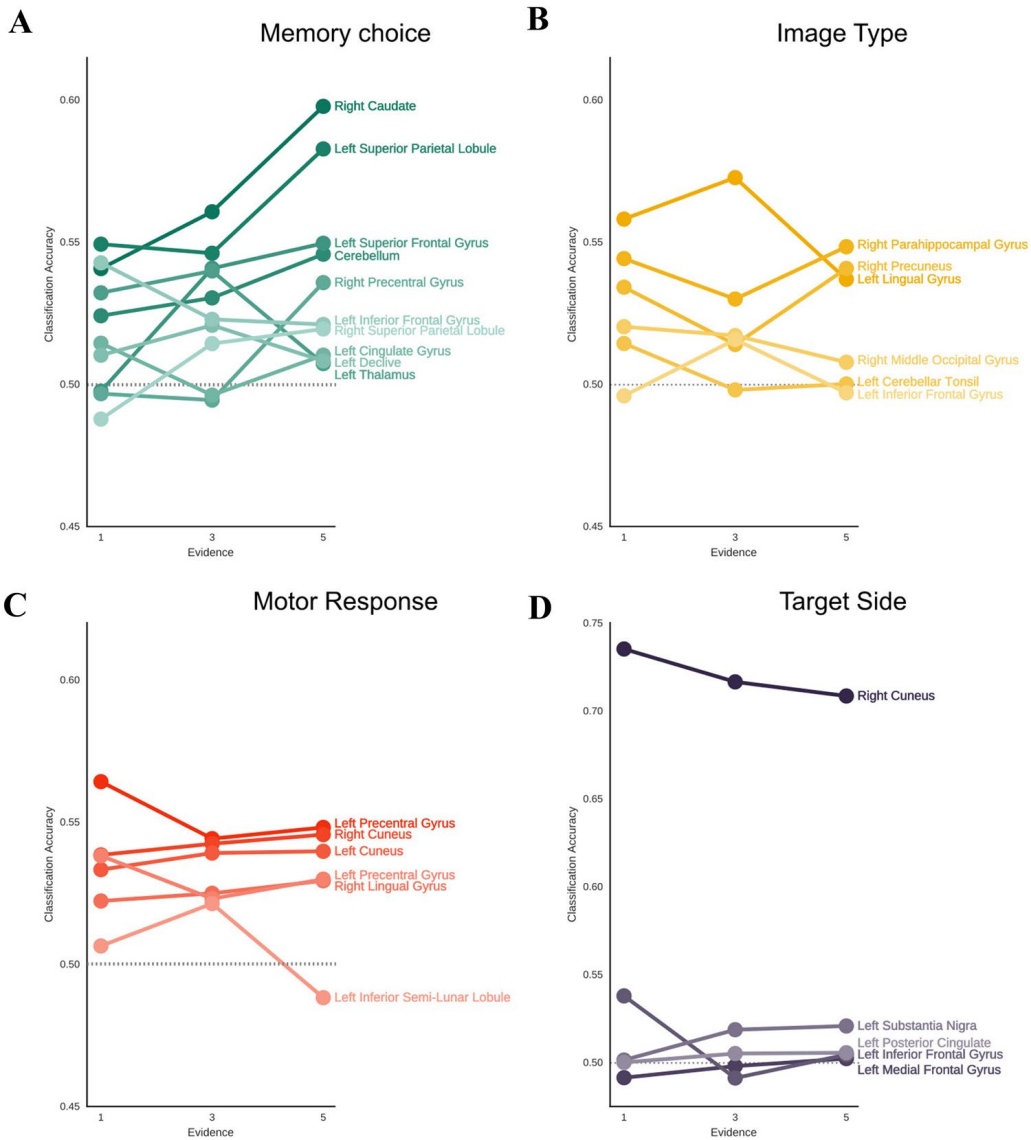
Modulation of memory evidence in task-related ROIs. The figure shows the decoding accuracy (*y*-axis) as a function of memory evidence (*x*-axis) in the four sets of ROIs defined by the between-subject searchlight analysis: memory choice (green), motor response (red), image type (yellow), target side (violet). Each line represents a ROI. Darker color indicates larger clusters/ROIs

Figure 2 shows the predictive accuracy for each task-related process in the corresponding sets of ROIs as a function of decision evidence. Statistical analyses (see Suppl. Table 1) demonstrated the presence of a significant linear increase in choice classification accuracy as function of decision evidence, analogous to the pattern observed in the behavioral results, in two clusters that showed choice-predictive activity: the right caudate (*p* < 0.01, corrected) and the left superior frontal gyrus (SFG, *p* < 0.05, corrected). Other clusters in bilateral PPC and in the right PFC showed a significant modulation (*p* < 0.05) but the effect did not survive correction for multiple comparisons. Consistent with a role in the memory decision, the right caudate exhibited a significant choice-predictive activity at the lowest (1 ×) level of evidence (*p* < 0.05, corrected), when the objective memory status and subjective decisions were considerably divergent and task performance was near chance level (see behavioral results). The same result was observed in the left PPC (*p* < 0.05, corrected) and in the left inferior frontal gyrus (IFG, *p* < 0.05, corrected), but not in the left SFG (*p* = n.s.). Importantly, decoding of the memory choice in the right caudate performed significantly better than decoding of the memory status (paired t test, *p* < 0.05, corrected). A similar result was only observed for the left PPC (*p* < 0.05) albeit it did not survive the correction for multiple comparisons.

In addition, consistent with our prediction, increasing decision evidence did not produce a parallel increase of classification accuracy in any of the clusters associated with decoding of the image type (Fig. 2b, all ROIs *p* = n.s.). Crucially, also the clusters associated with action-predictive activity were insensitive to the manipulation of decision evidence (Fig. 2c, all ROIs *p* = n.s.). Similar results were observed for the clusters associated with decoding of the target side (Fig. 2d, all ROIs *p* = n.s.). The only exception is represented by a cluster in the left IFG, which exhibited a significant modulation, although it was in the opposite direction and did not survive the correction for multiple comparisons. Overall, the present analysis demonstrated that the manipulation of decision evidence selectively modulated choice-predictive activity in striatal, left prefrontal and, to a lesser extent, in left parietal and right prefrontal regions, paralleling the parametric effect on behavioral performance. Instead, the manipulation had no effect on action-predictive activity, supporting the independence between brain signals related to memory decisions and motor intentions.

### Temporal decoding of choice-predictive and action-predictive activity

**Fig. 3:**
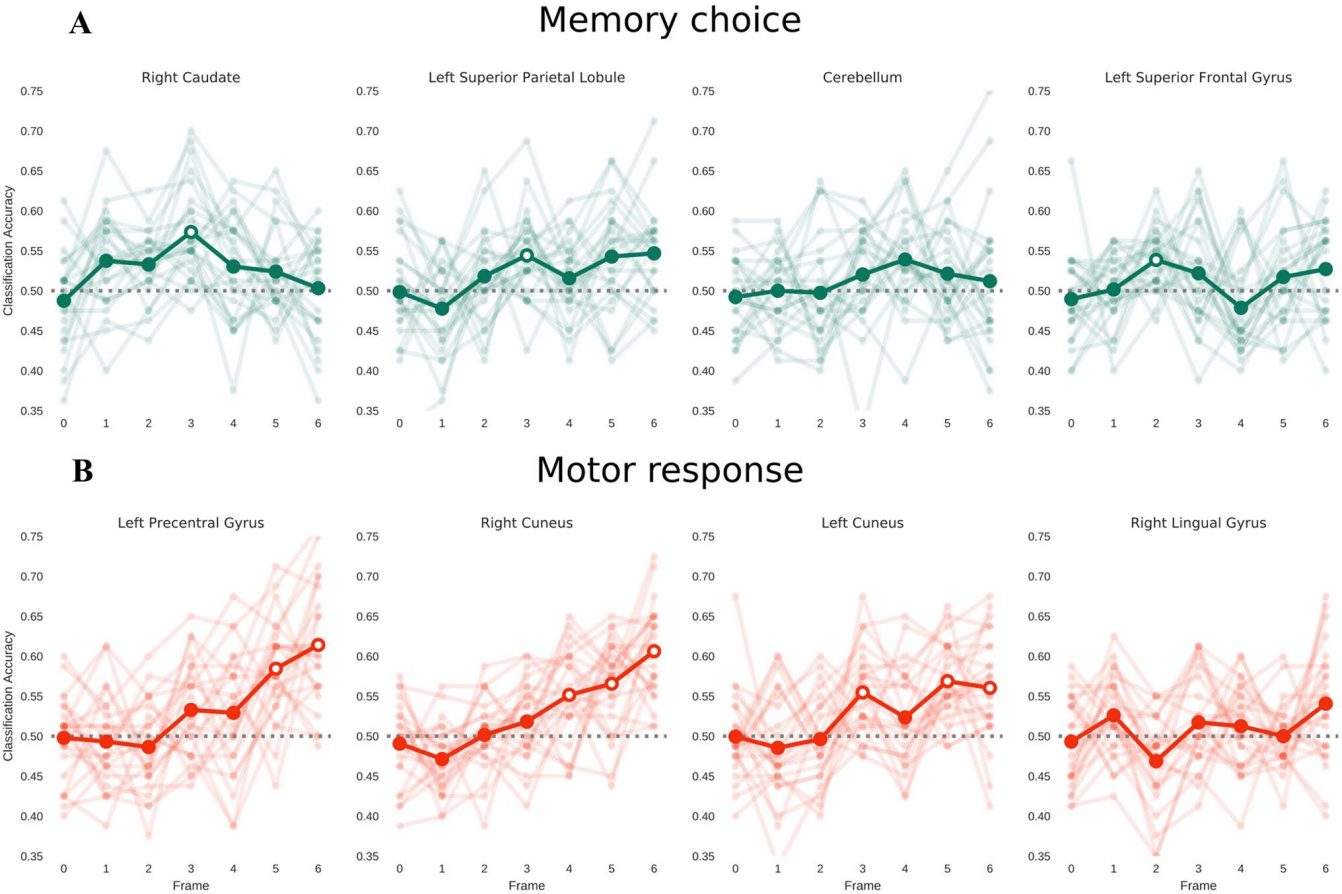
Temporal decoding of choice- and action-predictive activity. The figure shows the temporal profile of decoding accuracy for choice-predictive (green) and action-predictive (red) activity. The ROIs with the largest classification accuracy in the searchlight analysis are shown. Time-points reaching significant decoding accuracy are highlighted as white dots in the time-course of activity

We further examined the time-course of classification accuracy using a between-subject temporal decoding analysis. Of interest was to determine the temporal profile of decoding activity in choice- and action-predictive clusters to evaluate their similarity. We automatically identified peaks in the average temporal decoding signal using an approach that finds all local maxima in the signal by comparisons with neighboring values. The analysis of choice-predictive activity indicated an early peak of modulation at frame 3 (Fig. 3a), with significant decoding (*p* < 0.05, corrected) obtained in the middle of the decision phase, ranging from 4 s from the image onset in the left superior frontal to 6 s from image onset in the right caudate and left PPC, respectively. Notably, classification accuracy of choice-predictive activity (Fig. 3b) showed a transient profile, decaying right after reaching the statistical significance (see Suppl. Fig. 1 for results in the whole set of ROIs). The modulation of action-predictive activity, instead, showed a much more gradual increase, reaching a statistical significance (*p* < 0.05, corrected) only at the end of the decision phase in the left precentral gyrus (approx. 10 s from image onset) and in the right cuneus (approx. 8 s from image onset) and remained sustained until the go signal for movement execution. In addition, the peak identification algorithm found only a local maximum at frame 6. An exception to this delayed pattern of modulation was represented by the profile of the left cuneus, in which significant classification was also observed in an early phase of the decision phase (around 6 s from image onset). Overall, this analysis indicates that choice- and action-predictive activity across subjects showed distinct temporal profiles during the delay period, with generally early transient choice-predictive signals preceding late, sustained action-predictive signals, as also demonstrated by the different frame of peak occurrence.

### Temporal decoding of subject-specific signals associated with the choice–response association

The previous analyses focused on the identification of choice- and action-predictive signals that were consistently observed across subjects, regardless of the particular choice–response association, demonstrating that the two signals strongly differ both in terms of temporal profile and sensitivity to decision evidence. As stated in the introduction, however, a central question of the present work was to investigate potential mechanisms mediating the transformation of decision-related signals into appropriate actions. To this aim, we exploited the collinearity between choice- and action-predictive signals to identify decision-related activity that was distinctive of the specific choice–response association provided to subjects at the beginning of the experiment. Said differently, since the association between memory choices and motor responses was fixed within, but not between, subjects, the specific pattern mediating the memory-response transformation could emerge by examining within- and between-subject differences.

**Fig. 4:**
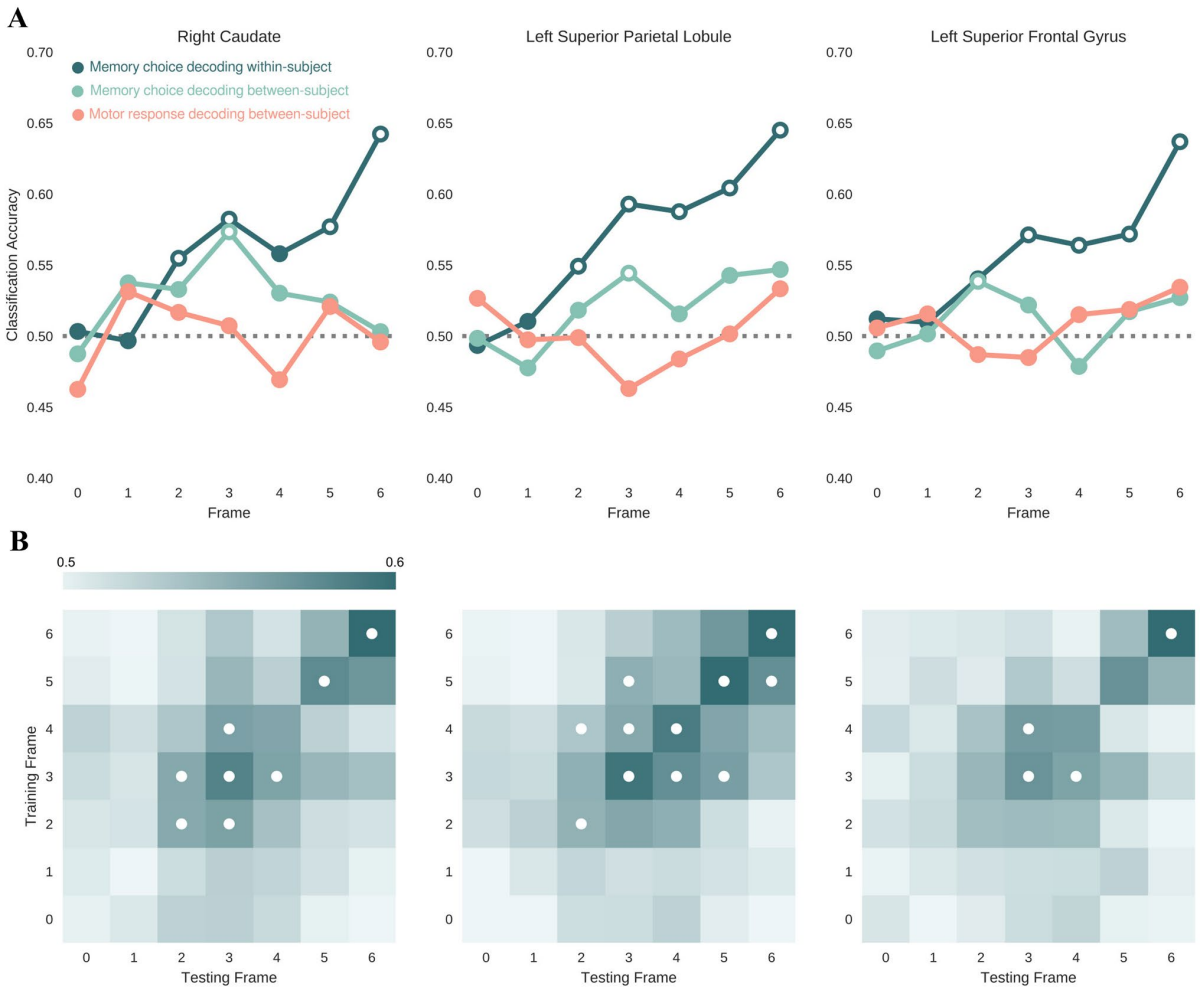
Temporal decoding and generalization of within-subject analysis. The upper row shows the temporal profile of within-subject decoding of decision-related activity (gray line), and of the between-subject choice-related (green line) and action-related (red line) activity. Only the ROIs with significant between-subject choice-predictive classification accuracy in at least one time point (light green) are shown. The time-points with significant decoding accuracy are highlighted as white dots in the time-course of activity. The bottom row represents the temporal generalization accuracy matrix, for each ROI, using the within-subject decision decoding. Statistical significance of the classification is indicated by black dots

We, therefore, tested whether regions exhibiting significant early, transient choice-predictive activity and modulation by decision evidence (i.e. caudate nucleus, left prefrontal and left parietal) also exhibited some features of such putative transitional signals. Consistent with the results of the between-subject design, in the cluster corresponding to the caudate nucleus (Fig. 4a, left panel) the peak detection algorithm found a first peak of within-subject classification accuracy (dark green line) at frame 3, which corresponds to the peak of across-subject choice-predictive activity (light green line). However, the algorithm also identified a second peak of within-subject classification accuracy at frame 6, which corresponds to the end of the decision period. Importantly, the between-subject analysis of action-predictive activity in the same regions indicated the absence of significant classification accuracy (light red) up to the end of the decision period. Therefore, the second peak of within-subject classification accuracy could not be explained by a corresponding peak of action-predictive activity across-subjects and likely reflects a signal that is highly dependent on the particular choice–response association. A similar second peak of decision-related activity was identified when conducting within-subject analyses in the parietal and prefrontal clusters (Fig. 4a, middle and right panel), although the divergence between the two analytic approaches was less striking compared with the striatal cluster (see Suppl. Fig. 2A for results in the whole set of ROIs).

We further investigated the nature of this second bump of decision-related activity using the temporal generalization method. In particular, to test whether the distributed pattern of choice-predictive activity is stable or changes during the decision period, we evaluated the ability of a decoder, trained on a specific time point, to accurately classify activity on different time points. To this aim, we tested whether the classifier trained on patterns at first peak (frame 3) could accurately decode the activity in the second peak (frame 6), a result that would indicate a temporally stable neural code. We found that the pattern of activity observed in the first peak (frame 3) is very similar to those of adjacent frames (frame 2 and 4; *p* < 0.05; corrected) but decreased in frame 5 and 6, suggesting the presence of a different neural code. The results indicated a scarce temporal generalization for both the striatal and the prefrontal clusters, suggesting that the two processes are mediated by distinct neural codes (Fig. 4b). A similar, albeit less clear, temporal distinction was observed in the left PPC cluster (see Suppl. Fig. 2B for results in the whole set of ROIs). To summarize, the second peak of decision-related activity observed in the within-subject analysis does not reflect pure choice-predictive activity nor pure action-predictive signals, but rather an intermediate decision signal that is strongly dependent on the actual choice–response association.

## Discussion

In the present work, we examined the relationship between signals associated with memory decisions and motor intentions in the human brain. Using a MVPA approach and a paradigm that manipulated the association between a memory decision (i.e. old, new) and a specific motor response (i.e. hand, eye movement) across subjects, we identified locally distributed activity that predicts memory choices and motor intentions independently from the decision–response association. Choice-predictive activity was mainly observed in striatal, lateral prefrontal and lateral parietal regions and showed a rapid increase after stimulus onset, followed by a fast decay. Notably, in the striatum, and to a lesser extent in lateral frontoparietal clusters, choice-predictive activity was significantly modulated by the amount of decision evidence. Thus, the multivariate pattern of brain activity fits closely with the parametric effect of the manipulation on behavioral performance. Moreover, choice-predictive activity remained significant also when the subject’s performance almost approached chance level, as indicated by the behavioral analysis. Action-predictive signals, examined before movement execution, were found in primary sensory-motor, premotor and occipito–parietal regions that overlap with known effector-specific regions of the human cortex. Classification of motor intentions was generally observed at the end of the decision phase and was not modulated by the amount of decision evidence. These findings are consistent with the involvement of a fronto–striato–parietal network in the early representation of the decision variable, which, however, is not directly transformed into an action plan. Finally, we addressed the question of the putative conversion mechanism of memory decisions into actions. Specifically, we investigated whether such transformation can be indirectly implemented in choice-predictive regions through the expression of an intermediate decision signal that, unlike pure choice- and action-predictive signals, depends on the particular choice–response association. Consistent with the hypothesis, we observed a second, delayed peak of decision-classification accuracy, most evident in the striatum that might represent a neural code of such transitional signal.

### Fronto–parietal regions involved in memory decisions

**Fig. 5:**
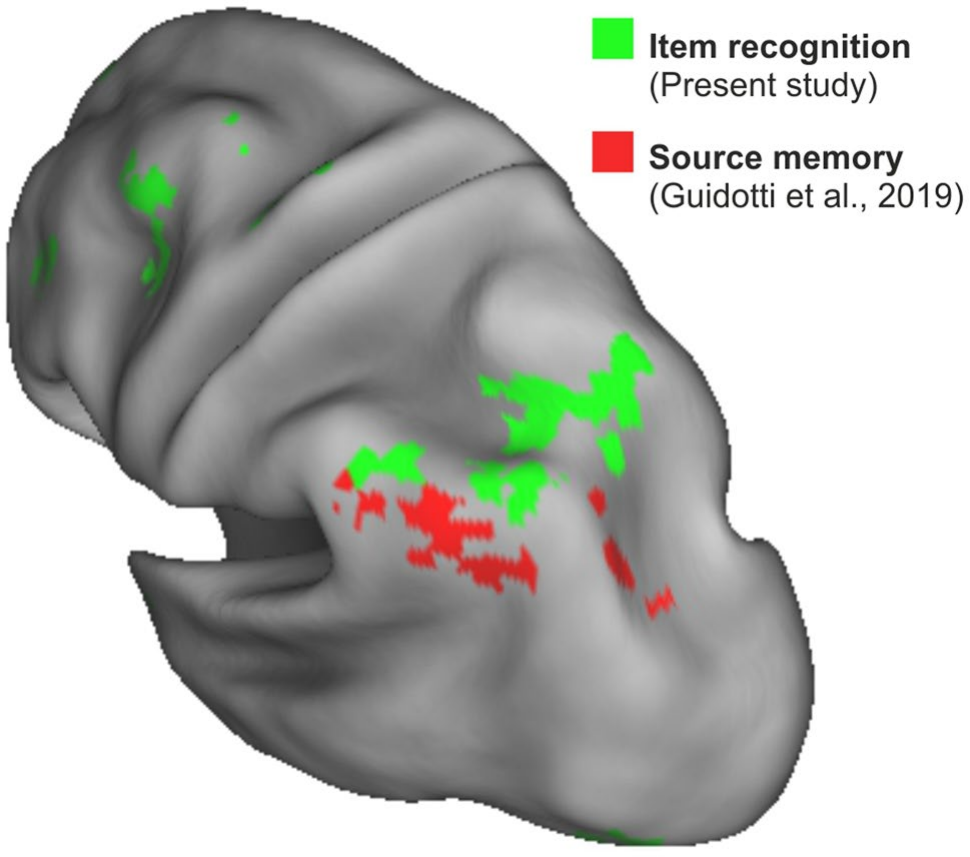
Choice-predictive activity in item recognition and source memory. The figure illustrates the topographical relationship in parietal regions between choice-predictive activity during item recognition (green, present study) and source memory [red, (Guidotti et al. 2019)]. The two studies used very similar across-subject MVPA approaches

The seminal observation that BOLD activity in the left parietal cortex tracks perceived oldness regardless of the accuracy of item recognition (Wheeler and Buckner 2003; Kahn et al. 2004) has led to the hypothesis that this portion of the cortex, and especially areas located in the proximity of the intraparietal sulcus, are involved in accumulating evidence for memory-based decisions (Wagner et al. 2005). We have provided further evidence that the profile of activity in this parietal region, but also in prefrontal and striatal regions, is compatible with the representation of a decision variable during both item recognition (Sestieri et al. 2014) and source memory (Guidotti et al. 2019) decisions. The present results, obtained through a reanalysis of our original item recognition dataset, provide further evidence for the presence of decision-related activity in parietal and frontal regions and allow a comparison of the spatial distribution of choice-predictive signals across studies that used a similar between-subject MVPA approach but different memory paradigms (Fig. 5). Interestingly, choice-predictive activity during item recognition and source memory is observed in regions, respectively, located more dorsally and more ventrally with respect to the intraparietal sulcus. This topographical distinction might reflect the greater contribution of familiarity and recollection signals in the two types of decisions and is consistent with previous evidence suggesting that these processes are associated with more dorsal and ventral parietal activations (Vilberg and Rugg 2008; Wagner et al. 2005). We note, however, that several features of the present multivariate approach (e.g. the classification of decision outcome regardless of decision accuracy, the modulation by decision evidence) likely emphasized the contribution of post-retrieval signals associated with decision-making rather than mnemonic signals directly involved in the retrieval process.

The present results additionally demonstrate that, when directly testing whether regions track the memory decision or the action intention, decision signals seem to predominate in the left lateral posterior parietal cortex. The fact that action-predictive activity was only observed in the most ventral aspect of the medial PPC is perhaps surprising, given the known role of the parietal cortex in motor planning across different task paradigms (Andersen and Cui 2009). As a matter of fact, several studies have suggested that the PPC comprises a mosaic of multiple intentional maps, each specialized for a particular motor effector based on its pattern of cortico-cortical connections (Andersen and Cui 2009; Rizzolatti et al. 1997). These sensori-motor regions are also involved in the allocation of visuospatial attention and are predominantly located in the posterior-dorsal aspect of the lateral PPC and in the medial PPC (Astafiev et al. 2003; Connolly et al. 2003; Galati et al. 2011; Sereno et al. 2001). However, effector-specificity is far from absolute, especially when focusing on signals that precede movement execution, and the location of effector-specific regions show considerable inter-individual differences (Filimon 2010; Galati et al. 2011; Gallivan and Culham 2015; Heed et al. 2011). The present work, which was based on a between-subject design that specifically highlights consistent patterns of activity across subjects, might in part account for the absence of action-predictive activity in left lateral PPC. At the same time, the inter-subject variability of action predictive signals appears in contrast with the shared spatial representation of retrieval-related activity, which has been demonstrated across different paradigms (Chen et al. 2017; Guidotti et al. 2019; Kragel and Polyn 2016).

Finally, the present study shows that regions involved in action planning, i.e. that are sensitive to the effector used to report the old/new decisions, were not modulated by the amount of decision evidence and were thus insensitive to a key decision variable that had a profound effect on behavioral performance. This seems to be the case regardless of whether the sensori-motor regions are defined a priori using an active localizer task (Sestieri et al. 2014) or, as in the present study, by focusing on action-predictive signals. In the field of perceptual decision-making, such modulation has been often interpreted as a sign of the substantial overlap between the neural system supporting decision and action planning. We note that the present results do not rule against the presence of multiple potential plans in sensori-motor regions before movement execution (Cisek and Kalaska 2005), but suggest that evidence for recognition decision is not directly translated into action plans, as in the case of sensory evidence during perceptual decisions (Donner et al. 2009; Gould et al. 2012; Tosoni et al. 2008). This apparent lack of embodiment might reflect the fact that memory-based decisions are associated with general intentions to act but not with the use of a specific effector.

### Multiple roles of the striatum in memory-based decisions

Recent neuroimaging and neuropsychological evidence have contributed to expand the view of basal ganglia involvement beyond non-declarative memory [reviewed in Scimeca and Badre (2012)]. The emerging view is that the striatum is not the source of the mnemonic signal itself (i.e. it is not involved in the actual retrieval of information) but it may act to modulate the memory retrieval process in the service of the current behavioral goals. Several findings of the present study converge to highlight the involvement of the striatum in different aspects of memory-based decisions. In particular, locally distributed activity in this region showed the highest choice-classification accuracy and sensitivity to decision evidence and significantly predicted choices also in the lowest evidence condition, when perceived oldness and objective memory status were most divergent (see Abe et al. 2008). Furthermore, the within-subject analysis, which does not distinguish between choice- or action-predictive activity, suggests that this region might be crucial for representing the association between memory decisions and motor commands. Indeed, whereas the first peak of classification accuracy was representative of decision-related activity that was shared across subjects, the second peak could not be associated neither with choice- nor with action-predictive activity observed across subjects. We acknowledge that multiple sources of inter-subject variability (i.e. idiosyncratic spatial and temporal features of locally distributed activity) might explain the lower performance of the between-subject compared to the within-subject analyses. However, we believe that the main factor that contributed to the observed difference between the results of the two decoding approach is the manipulation of the choice–response association. The lack of temporal generalization between the two peaks of activity suggests that the second peak reflected a different neural code or the involvement of a distinct neural population.

The first peak of decision-predictive activity observed in the present study, reflecting the differential classification of old vs. new items, is consistent with the proposed role of the striatum in signaling the adaptive significance of perceived oldness for the current goals (Scimeca and Badre 2012). Previous evidence indicates that old items are intrinsically associated with higher reward than new items especially when successful retrieval happens in difficult recognition tasks (Bunzeck et al. 2010; Han et al. 2010). On this basis, one might assume that the early peak of classification accuracy reflects the contribution of the striatum in the evaluation stage of the decision process, which is associated with the representation of the subjective values of choice options (Kable and Glimcher 2009).

Instead, the second peak of classification activity observed in the striatum appears to represent the transformation of a memory decision signal (old/new) into a neural code that helps specifying what to do with the output of the memory decision (i.e. the appropriate action). This information could be in turn used by regions involved in the execution of the motor plan (eye/hand), which do not directly represent a memory decision variable. This function is likely performed in combination with fronto-parietal regions, but we note that the distinction between the two peaks of decision-predictive activity is most evident in the striatum. The basal ganglia have been strongly associated with the control of habits (e.g. Rangel et al. 2008). Specifically, it has been argued that the habit system learns to assign values to actions and stimulus–response associations in stable environments. One interpretation of the current results is, therefore, that the striatum is involved in the conversion process from the presumably biased (old vs. new) evaluation system to the creation of the stimulus–response bonds (which indicate the action that should be taken in a particular state of the world) that maximize reward. In summary, by following the framework of the ‘actor–critic’ architecture proposed by Sugrue and colleagues (Sugrue et al. 2005), we propose that the striatum would be both actively involved in the valuation stage of assignment of subjective value of the choice option (actor, first peak of classification accuracy) and in the creation of the optimal stimulus–response association (critic, second peak).

An additional interpretation of the second peak of classification accuracy in the striatum is based on the well-known role of this structure in action selection. As extensively described by studies on functional anatomy of the striatum (Redgrave et al. 2010, 2011), this structure receives direct and indirect excitatory inputs from the cortex and the brainstem, respectively, and returns output information through inhibitory signals (again both directly and indirectly) to the cortex and the thalamus. Such a re-entrant looped architecture, allowing selective removal of tonic inhibitory output on specific loops, would represent an optimal neural basis for the implementation of action selection (Redgrave et al. 2011). Thus, according to this view, the second peak of activity in the striatum would directly reflect response selection rather than the specification of the memory choice/response association.

The comprehension of the role of the striatum in memory decision-making is also relevant for clinical neuropsychology investigations, especially for the understanding of the behavioral pattern of patients with a known dysfunction of the nigra-striatal pathway (e.g. Parkinson disease). Previous research has shown that these patients do not usually suffer from profound amnesia but can exhibit multifaceted recognition deficits (see Scimeca and Badre 2012). Based on the present results, we predict that these patients would not be particularly impaired in recognition per se, but rather in assigning the adaptive significance of oldness and newness to the current goal/context, or in producing specific responses as a function of the recognition decision. These deficits might be exacerbated by paradigms that manipulate, within subjects, which option is incentivized, or which action must be executed based on the memory choice.

## Electronic supplementary material

Below is the link to the electronic supplementary material.

Supplementary file1 (PDF 436 kb)
