# Supplementary material for: Properties and temporal dynamics of choice- and action-predictive signals during item recognition decisions
Source: Brain Struct Funct. 2020 Aug 9;225(7):2271–86. doi: 10.1007/s00429-020-02124-4 (PMC7473849; doi:10.1007/s00429-020-02124-4)
Supplement: Supplementary file 1 — Supplementary file1 (PDF 436 kb) [file 429_2020_2124_MOESM1_ESM.pdf]

| Task-related information | Region | Effect of evidence | | |
|---|---|---|---|---|
| | | T | p | p corr |
| Memory choice | Right Caudate | 3,714 | **0,001** | **0,005** |
| | Right Superior Parietal Lobule | 2,080 | **0,043** | 0,430 |
| | Left Superior Parietal Lobule | 2,186 | **0,034** | 0,338 |
| | Right Pyramis | 1,624 | 0,111 | 1,110 |
| | Left Superior Frontal Gyrus | 3,139 | **0,003** | **0,029** |
| | Left Thalamus | -1,627 | 0,111 | 1,105 |
| | Right Precentral Gyrus | 2,788 | **0,008** | 0,076 |
| | Left Cingulate Gyrus | -0,248 | 0,806 | 8,055 |
| | Left Declive | -0,127 | 0,899 | 8,994 |
| | Left Inferior Frontal Gyrus | -1,469 | 0,148 | 1,484 |
| Motor response | Left Precentral Gyrus | -1,009 | 0,318 | 1,909 |
| | Right Cuneus | 0,475 | 0,637 | 3,822 |
| | Left Cuneus | 0,412 | 0,682 | 4,094 |
| | Right Lingual Gyrus | 0,506 | 0,616 | 3,693 |
| | Left Precentral Gyrus | -0,452 | 0,653 | 3,920 |
| | Left Inferior Semi-Lunar Lobule | -1,282 | 0,206 | 1,237 |
| Target side | Right Cuneus | -2,002 | 0,051 | 0,255 |
| | Left Medial Frontal Gyrus | 0,864 | 0,392 | 1,959 |
| | Left Inferior Frontal Gyrus | -2,159 | **0,036** | 0,180 |
| | Left Substantia Nigra | 1,501 | 0,140 | 0,700 |
| | Left Posterior Cingulate | 0,395 | 0,695 | 3,474 |
| Image type | Left Lingual Gyrus | -1,396 | 0,169 | 1,016 |
| | Right Parahippocampal Gyrus | 0,384 | 0,702 | 4,215 |
| | Right Precuneus | 0,507 | 0,614 | 3,686 |
| | Left Cerebellar Tonsil | -1,151 | 0,256 | 1,534 |
| | Right Middle Occipital Gyrus | -1,090 | 0,281 | 1,689 |
| | Left Inferior Frontal Gyrus | 0,081 | 0,936 | 5,616 |

**Table S1: Results of statistical analyses on the linear effect of evidence.**
The first column indicates the task-related information that was classified. The second column indicates the region name (see also Table 1). The other columns indicate the statistical values of Linear Mixed Model test on the linear effect of evidence in classification accuracies: the t-value, the corresponding uncorrected p-value and the p-value corrected for multiple comparisons (using Bonferroni correction for the number of clusters in each set). Values in bold are statistically significant according to $p<0.05$.

| Task-related information | Region | Classification accuracy (low evidence cond.) | | | Memory choice vs. status (1x) | | |
|---|---|---|---|---|---|---|---|
| | | T | p | p corr | T | p | p corr |
| Memory choice | Right Caudate | 3,667 | **0,001** | **0,013** | 3,458 | **0,001** | **0,011** |
| | Right Superior Parietal Lobule | -1,032 | 0,313 | 3,129 | -0,509 | 0,613 | 6,131 |
| | Left Superior Parietal Lobule | 4,442 | **0,000** | **0,002** | 2,031 | **0,048** | 0,480 |
| | Right Pyramis | 2,082 | **0,049** | 0,486 | 0,605 | 0,548 | 5,480 |
| | Left Superior Frontal Gyrus | -0,205 | 0,839 | 8,391 | -0,560 | 0,580 | 5,798 |
| | Left Thalamus | 2,078 | **0,049** | 0,491 | 1,445 | 0,155 | 1,551 |
| | Right Precentral Gyrus | -0,279 | 0,782 | 7,824 | 0,634 | 0,529 | 5,290 |
| | Left Cingulate Gyrus | 1,339 | 0,194 | 1,935 | 0,327 | 0,745 | 7,452 |
| | Left Declive | 0,969 | 0,342 | 3,424 | 0,207 | 0,837 | 8,367 |
| | Left Inferior Frontal Gyrus | 4,501 | **0,000** | **0,002** | 1,090 | 0,282 | 2,815 |

**Table S2: Classification of memory choice in the condition with the lowest level of decision evidence.**
The first column indicates the task-related information that was classified. The second column indicates the region name. The next three columns indicate the result of the one-sample t-test of classification accuracy vs. chance level performed in the condition characterized by the lowest amount of decision evidence: t-value, uncorrected p-value and p-value (bonferroni) corrected. The last three columns indicate the result of the paired t-test between the classification of memory choice and memory status in the same condition. Values reported in bold are statistically significant according to p<0.05.

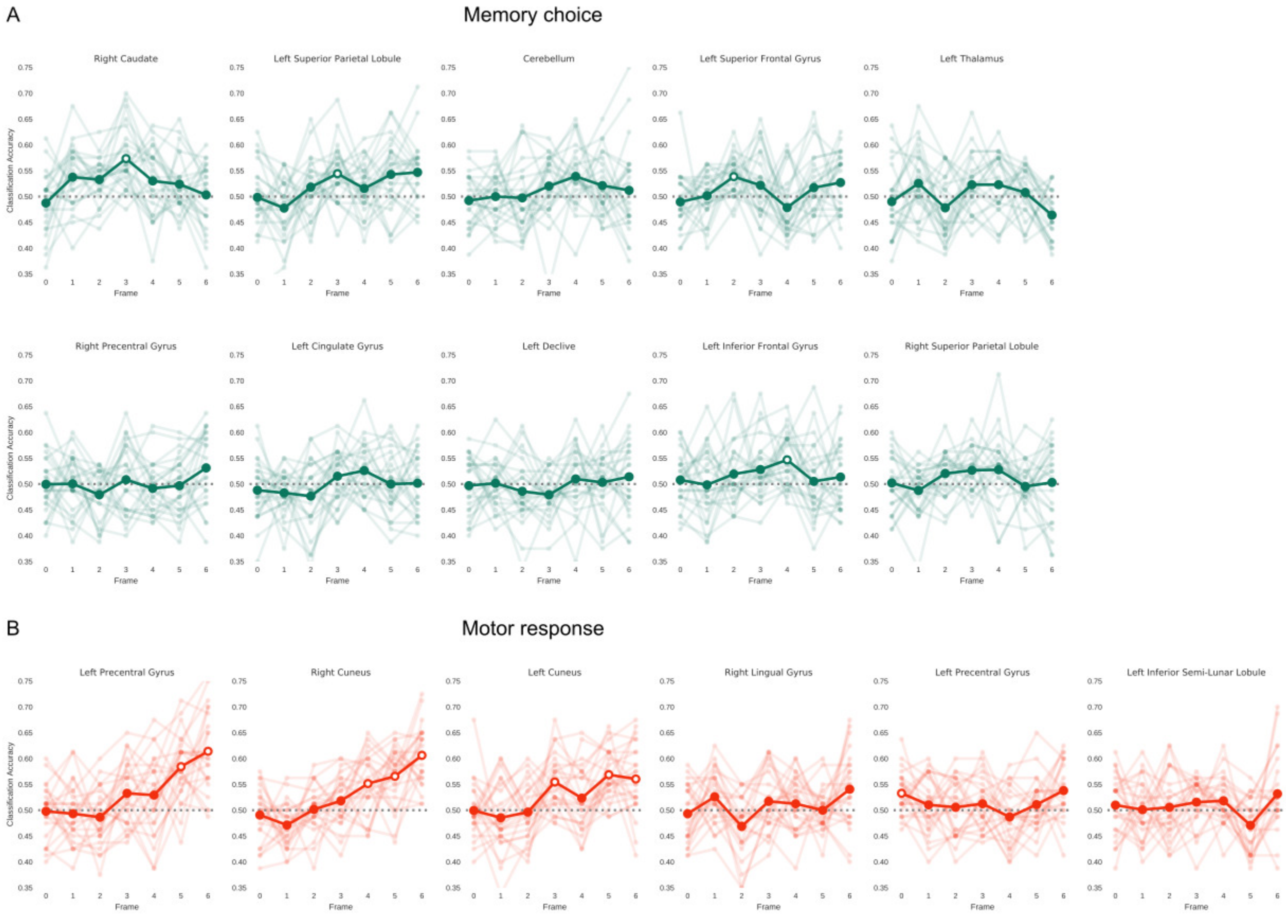


**Figure S1: Temporal decoding of choice- and action-predictive activity.**

The figure shows the temporal profile of decoding accuracy in all the ROIs identified using the searchlight analysis (see Table 1), for choice-predictive (panel A, green) and action-predictive (panel B, red) activity. Time-points with significant decoding accuracy are highlighted as white dots.

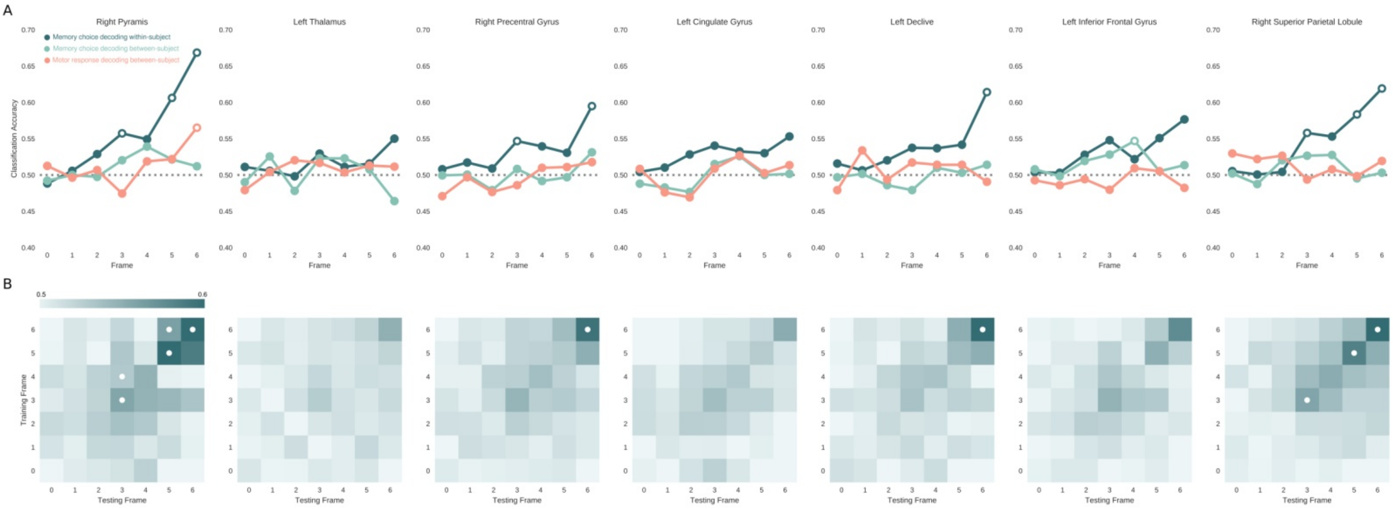


**Figure S2: Temporal decoding and generalization of within-subject analysis.**

**Panel A** illustrate the temporal profile of decoding accuracy for within-subject decision-related activity (teal-blue), and for between-subject choice-predictive (green) and action-predictive (red) activity in those ROIs with significant choice-predictive activity (Table1) that were not presented in Figure 4. The time-points with significant decoding accuracy are highlighted as white dots in the time-course of activity.

**Panel B** illustrates the temporal generalization accuracy matrices obtained with the within-subject memory decision decoding in the same regions. Statistical significance of the classification is indicated by white dots.
